# Isoniazid hair drug levels among TB patients as a tool to monitor adherence, exposure, and TB treatment outcomes and its acceptability in a multicultural setting. A narrative review

**DOI:** 10.4314/ahs.v23i4.5

**Published:** 2023-12

**Authors:** Grace Muzanyi, Muhammad Ntale, Robert Salata, Moses Joloba, Jackson Mukonzo, David Mafigiri, Paul Mubiri, Godfrey Bbosa

**Affiliations:** 1 Department of Pharmacology and Therapeutics, Makerere College of Health Sciences; 2 Uganda-Case Western Reserve University Research Collaboration; 3 Makerere University School of Public Health; 4 Case Western Reserve University, Cleveland Ohio; 5 Makerere University School of Social Sciences; 6 Makerere University School of Biomedical Sciences; 7 Department of Chemistry, College of Natural Sciences, Makerere University

**Keywords:** TB patients, adherence, TB treatment outcomes, multicultural setting, narrative review

## Abstract

**Background:**

Accumulation of chemicals including drugs in hair has been used in forensic investigations. Studies have reported isoniazid drug levels in the hair of TB patients.

**Objective:**

To review literature for evidence on isoniazid hair drug levels as a tool to monitor adherence, exposure, and TB treatment outcomes and the acceptability of using human hair for medical testing.

**Methods:**

We reviewed literature through Pubmed, Embase, Medline, google scholar, and google grey literature. The search terms focused on isoniazid/TB hair drug levels, adherence, treatment outcomes, and acceptability of using hair for medical testing. We kept refining our search terms at each step of our search.

**Results:**

The initial search yielded 186859 articles, which dropped to 88 after removing duplicates and irrelevant articles and eventually to 14 on further refining our search terms. On full review,2 out of 14 and 1 out of 14 articles touched the area of Isoniazid hair drug levels; adherence, exposure, TB treatment outcomes, and acceptability respectively. Further scrutiny showed that none of the articles had addressed our research question.

**Conclusion:**

Literature on Isoniazid hair drug levels among TB patients as a tool to monitor adherence, exposure, and TB treatment outcomes, and its acceptability is limited and more research is needed.

## Introduction

Hair is a key part of the human body that completes human creation. Human scalp hair is reported to grow at a rate of about 1 cm per month and drugs, chemicals, etc. are known to accumulate in hair with a time component added. Studies^1^ have demonstrated the accumulation of isoniazid in the hair of MDR, and Latent TB-infected patients. Studies have also shown the accumulation of antiretroviral drugs in the hair of HIV^2^ patients and these hair drug levels have been used to monitor adherence to HIV treatment. Hypotheses explaining the drug accumulation in the hair of TB patients are central to the exploration and subsequent use of isoniazid hair drug levels to monitor adherence, exposure, and predict treatment outcomes of TB patients. In addition, the harvest of human hair is a sensitive matter and has been associated with attempted witchcraft in African traditional society.

Of late, hair has been found to bear diagnostic and prognostic purposes in the world of medical practice. Several studies^2^-^3^ have been conducted on assessing hair drug levels of different components including TB drugs. TB treatment adherence and exposure are key determinants of the outcomes of TB treatment. Unfortunately, albeit the availability of drugs, some cases end up as treatment failure or relapse/drug resistance^4^-^5^ resulting from poor adherence, and exposure. In these situations, the patient experiences a poor quality of life. The World Health Organization recommends directly observed therapy but this is only feasible for inpatients yet the majority of TB patients are treated as outpatients and it does not monitor exposure and predict treatment outcomes. There are studies^3^, ^6^ about the use of plasma, saliva, and urine drug levels as a tool to monitor TB treatment adherence and exposure but these require daily sampling which is not convenient for patients and health workers. Sputum culture can monitor TB treatment outcomes but it's expensive, has a long turnaround time, and can't be used to directly monitor adherence and exposure.

In the treatment of TB with first-line drugs, we lack a tool that can monitor exposure, adherence, and treatment outcomes at the same time. TB drugs are currently dosed as fixed-dose combinations and studying one drug in the combination equates to studying adherence, exposure, and treatment outcomes with regard to all drugs in the combination.

Isoniazid is one drug among the first-line TB drugs that has been extensively studied and the techniques of its assay in body compartments^7^ have been established. The current first-line anti-TB regimen is given as a combination of Isoniazid+Rifampicin+pyrazinamide+ Ethambutol for 2 months followed by a combination of Rifampicin+ Isoniazid for 4 months^8^-^9^. We can note that Isoniazid and Rifampin are given throughout the treatment phase of first-line TB treatment but Isoniazid quantification in different body compartments has been extensively studied^10^ and the methods are established. We, therefore, sought, to review, the literature to identify articles where isoniazid hair drug levels have been studied as a tool to monitor adherence, exposure, and treatment outcomes for first-lineTB treatment. We also aimed at exploring articles that studied the acceptability of using hair for medical testing among TB patients.

## Methods

We used the Boolean approach and reviewed the available literature through Pubmed search, Embase, and Google grey literature. Searching the Mesh terms Isoniazid OR TB hair drug levels, Isoniazid OR TB treatment adherence, Isoniazid OR TB treatment adherence measures, TB OR Isoniazid treatment exposure, first-line anti-TB OR Isoniazid treatment exposure, monitoring TB OR Isoniazid treatment adherence, monitoring TB OR Isoniazid treatment outcomes, hair test acceptability or compliance in therapeutic drug monitoring, Isoniazid TB hair drug levels and TB treatment outcomes, Isoniazid hair drugs levels and TB treatment exposure, isoniazid hair drug levels and TB treatment adherence, hair test acceptability and therapeutic drug monitoring, Isoniazid not TB hair drug levels,Isonizid not TB treatment adherence, Isonaizid not TB treatment exposure monitoring, isoniazid not TB treatment adherence. Abstracts from articles pertinent to our target research area were obtained. We looked at all articles with no language restriction. We filtered out the duplicates and selected the relevant articles.

We then further refined the search terms specifically focusing on our research area of interest “isoniazid hair drug levels as a tool to monitor adherence, exposure and treatment outcomes as well as acceptability of the use of hair for medical testing”. If a study pointed to the association of isoniazid hair drug levels to TB treatment exposure or adherence or treatment outcomes or acceptability of using hair for therapeutic drug monitoring, then this was fully analyzed. In addition, the bibliography and citations to the selected articles were also evaluated and relevant articles not previously found were also included in order to strengthen the search results. In addition, we searched Medline using the same search terms, and relevant articles were extracted. Eventually, key texts on isoniazid hair drug levels in relation to first-line multi-drug TB treatment exposure, adherence, outcomes, and acceptability of using hair for TB treatment monitoring, and the high-impact manuscripts on the subject of isoniazid hair drug levels were studied. We did further scrutiny, to find articles that touched on the area of Isoniazid hair drug levels among TB patients as a tool to monitor adherence, exposure, and TB treatment outcomes, and articles that addressed the issue of acceptability of hair harvest for medical testing.

## Results

**Figure 1:**
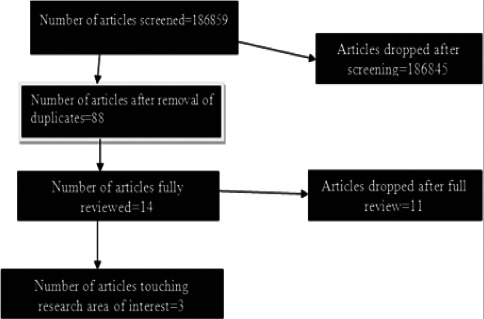
Study profile

We reviewed literature from 1956 through December 2023. We identified a total of 186859 articles initially but on further review, and removal of duplicates, the number of articles dropped to 88 (figure 1) that were relevant to our search terms (table 1) out of which 14 articles were eligible for a full review. Of the 14 articles fully reviewed, only 2(table 2) touched the research area of interest “Isoniazid hair drug levels among TB patients as a tool to monitor adherence, exposure, and TB treatment outcomes” and 1 touched the area of acceptability of hair testing for therapeutic drug monitoring but was not specific to TB patients. All the three articles touching on the research area of interest didn't address the research question of “Isoniazid hair drug levels among TB patients as a tool to monitor adherence, exposure, and TB treatment outcomes as well as acceptability of using hair for medical testing”.

**Table 1:** Articles yielded from the search terms

Search term	The number of articles (N)yielded
TB hair drug level	5
Isoniazid hair drug level	2
TB treatment adherence	77407
TB treatment adherence measures	50066
TB treatment exposure	3263
Isoniazid exposure	87
First-line anti-TB drug exposure	10
Monitoring TB treatment adherence	24926
Monitoring TB treatment outcomes	31074
Hair drug level and treatment outcomes	5
Isoniazid hair drug levels and Tb treatment outcomes	2
Isoniazid hair drug levels and TB treatment adherence	4
Isoniazid hair drug levels and TB treatment exposure	5
Acceptability of hair test for therapeutic drug monitoring	3
Acceptability of hair test for TB therapeutic drug monitoring	0
**Total**	186859

**Table 2:** Articles yielded after refining the search terms

Study	Year	Country	Sample size	Age group	Gender (Female)	Findings
Isoniazid hair drug levels, TB treatment exposure, and outcomes.	2021	India	264	<14yrs&>14yrs	50%	Hair levels of INH and its metabolite may predict TB treatment outcomes

Mave etal

Isoniazid hair drug levels and TB treatment adherence	2016	USA	18	3-76yrs	28%	Hair concentrations of isoniazid assess long-term exposure

Gerona etal

Acceptability of hair testing for therapeutic drug monitoring.	2021	Nigeria	398	40(SD±9.8)	64.8%	Two-thirds of the participants were willing to donate hair in Nigeria for biomedical testing.

### Isoniazid hair drug levels, TB treatment exposure, and TB treatment outcomes

Isoniazid hair drug levels defined as the quantity of isoniazid or its metabolites in a single hair tuft. TB treatment exposure is the quantity of TB drugs achieved in the body after drug administration. Mave et al (table 1) studied TB drug levels in the hair of adults and children to monitor drug exposure and treatment outcomes. In this study, about 30 strands of hair were cut from the occipital region in adults and children from a prospective TB cohort in India. Isoniazid (INH), acetyl-INH, and pyrazinamide (PZA) were extracted from the hair samples and quantified using liquid chromatography-tandem mass spectrometry. The relationship between drug concentrations in hair and time to unfavorable outcomes was assessed using Cox-proportional hazards regression models. He found that A two-fold increase in hair acetyl-INH concentrations indicated a lower hazard of unfavorable TB treatment outcomes (aHR 0.67, 95%CI 0.44-1.02) and TB treatment failure (aHR 0.65, 95%CI 0.42-1.01). Higher summed concentrations (a summed measure of INH and acetyl-INH) indicated a lower hazard of treatment failure (HR 0.69, 95%CI 0.45-1.05). He concluded that Hair levels of INH and its metabolite may predict TB treatment outcomes, indicating the potential utility of this measure to assess and optimize TB treatment outcomes.

### Isoniazid hair drug levels and TB treatment adherence

Treatment adherence is defined as the steady devotion by patients to take their TB treatment. Gerona et al (table 1) studied the quantifying of isoniazid levels in small hair samples for assessing adherence during the treatment of latent and active TB. In this study, a large hair sample from a patient with active TB was obtained for assay development. Methods to pulverize hair and extract isoniazid were optimized and then the drug was detected by liquid chromatography/ tandem mass spectrometry (LC/MS-MS). The method was validated for specificity, accuracy, precision, recovery, linearity, and stability to establish the assay's suitability for therapeutic drug monitoring (TDM). Hair samples from patients on directly-observe isoniazid-based latent or active TB therapy from the San Francisco Department of Public Health TB clinic were then tested. They found that LC/MS-MS-based assay detected isoniazid in quantities as low as 0.02ng/mg using 10-25 strands of hair. Concentrations in spiked samples demonstrated linearity from 0.05-50ng/mg. Assay precision and accuracy for spiked quality-control samples were high, with an overall recovery rate of 79.5%. In 18 patients with latent or active TB on treatment, isoniazid was detected across a wide linear dynamic range. They concluded that LC-MS/MS-based assay was developed and validated to quantify isoniazid levels in hair with performance characteristics suitable for TDM. Hair concentrations of isoniazid assess long-term exposure and may be useful for monitoring adherence to latent or active TB treatment in the setting of HIV.

### Acceptability of hair testing for therapeutic drug monitoring

Herbertson etal studied the acceptability of donating hair and other biological samples for research among people living with HIV in Nigeria. A cross-sectional survey of people living with HIV on ARV therapy (ART) was conducted at the HIV clinic of the Nigerian Institute of Medical Research, using systematic sampling. The researcher-administered questionnaire was designed to capture sociodemgraphic data, length of time on ART, and willingness to donate hair. Univariate analysis was performed on sociodemographic characteristics, and independent-sample t-tests and chi-square tests were used for bivariate analysis. Multivariable logistic regression analysis was performed to assess factors associated with willingness to donate hair samples, with a significance level of 0.05. Of the 398 participants enrolled in the study, 258 (64.8%) were female, the average age was 40 years (±9.8), and the average time spent on ART was 7.3 years (±4.2). More than half (64.8%) of the respondents were willing to donate hair samples for biomedical research and they were 1.5 times more likely to donate hair than blood. For one-third of the participants, the anticipated benefit from the eventual research findings was the primary motivation to donate hair samples. Fear of the use of hair for rituals was the most commonly stated reason for unwillingness to donate hair samples (21.2%). Nearly two-thirds of the participants were willing to donate hair samples for biomedical research in an ethnically diverse, urban-based Nigerian study population. These findings support the feasibility of hair sampling for future HIV clinical research conducted within Nigeria.

## Discussion

The use of hair for diagnostic, prognostic, and medical-legal investigations has become a subject of interest in recent years^11^-^12^. There is evidence supporting diagnostic and prognostic as well as medical legal roles of hair in modern times. In addition, hair is currently being used to predict underlying health conditions in the human body. There is a growing body of evidence on using hair for forensic investigations involving chemical poisoning^13^. In the HIV world, currently, hair drug levels of antiretroviral drugs are being used to monitor adherence to HIV treatment^1^. There have been studies on latent, MDR, and active TB to monitor exposure and adherence to treatment however, but our review has found that all these studies have stopped short of exploring the use of Isoniazid hair drug levels to monitor exposure, adherence, treatment outcomes and exploring whether this would be acceptable to the TB patient community in Uganda given the attribution of hair harvest to witchcraft.

Of the 14 articles filtered out of the 186859 articles in our review, only two articles touched the area of isoniazid hair drug levels for monitoring adherence, exposure, and treatment outcomes. Mave etal^2^ assessed hair levels of isoniazid, its metabolite, and Pyrazinamide and used them to predict outcomes of TB treatment, however, this study didn't reach statistical significance as evidenced by the confidence intervals that include the null value of 1 with the hazard ratio being the effect measure. He concluded that hair levels of INH and its metabolite may predict TB treatment outcomes, indicating the potential utility of this measure to assess and optimize TB treatment outcomes but this conclusion didn't come from the results he presented in this article since they are not supported by the confidence intervals. Also, this study didn't assess how TB sputum culture conversion relates to hair drug levels at 8 weeks of TB treatment. In addition, patient factors like weight, body mass index, alcohol consumption, smoking, and diabetes were not assessed how they affect isoniazid hair drug levels in this study. In addition, Mave et al didn't assess the relationship between isoniazid hair and plasma drug levels at a selected time point for a fixed isoniazid dose of 300mg in a patient who has achieved a steady state after at least 14 doses of TB treatment. The hair concentration at which TB sputum culture conversion occurs at 8 weeks as well the isoniazid hair drug level at which common side effects occur was also not assessed by Mave etal but this is very important to know as we can base on this to predict treatment outcomes. In addition, Mave etal didn't assess how Isoniazid drug levels differ in patients on DOT as opposed to patients on self-administration of drugs which reflects the pragmatic element of TB control programs where most patients do self-administration of drugs, particularly in resource-constrained settings like Uganda. Also this study was based in India which might raise questions if the finding s can be applied to Uganda/sub-Saharan Africa. Gerona etal^14^ conducted a study to assess isoniazid hair drug levels and TB treatment adherence during latent and active TB treatment in the USA. This study was specifically based in the first world setting where plenty of resources are available as compared to resource-limited settings like Uganda with limited resources. To generalize the findings in the Gerona study, a similar study has to be conducted in settings like Uganda. In the Gerona study, exposure and treatment outcomes were not assessed but only adherence. In addition, this study was about technique development and validation for isoniazid hair drug assays but he concluded that hair concentrations of isoniazid assess long-term exposure and may be useful for monitoring adherence to latent or active TB treatment in the setting of HIV. This conclusion does not come from the results presented in the article.

We didn't identify any article addressing acceptability of hair harvesting for medical testing among TB patients however, as a proxy to this area, Hebert son etal^15^ conducted a study to assess the acceptability of using hair for medical research testing among people living with HIV in Nigeria. The acceptability rates were high however, this study was not conducted among TB patients and this raises questions as to whether this result is reproducible or not when extrapolated to TB patients. The current literature has a dearth of studies addressing the question of the acceptability of hair harvest for medical testing among pulmonary TB patients.

In summary, these 3 articles that were selected in a relation to our area of interest “Isoniazid hair drug levels among TB patients as a tool to monitor adherence, exposure, and TB treatment outcomes as well as acceptability of using hair for medical testing among TB patients” didn't address this question. This leaves us with a literature gap to fill. We should therefore focus our efforts in this area to generate new knowledge.

## Recommendation

Literature on the use of isoniazid hair drug levels to monitor adherence, exposure, treatment outcomes as well as its acceptability in a multicultural setting like sub-Saharan Africa is limited. There's need for more research in this area to explore the potential utility of isoniazid hair drug levels in TB therapeutic drug monitoring and its acceptability given the traditional beliefs of witch craft associated with harvesting one's body hair.
